# CMR-based 3D statistical shape modelling reveals left ventricular morphological differences between healthy controls and arterial switch operation survivors

**DOI:** 10.1186/1532-429X-18-S1-Q2

**Published:** 2016-01-27

**Authors:** Jan L Bruse, Hopewell Ntsinjana, Claudio Capelli, Giovanni Biglino, Kristin McLeod, Maxime Sermesant, Xavier Pennec, Tain-Yen Hsia, Silvia Schievano, Andrew Taylor

**Affiliations:** 1 Centre for Cardiovascular Imaging - Great Ormond Street Hospital for Children, UCL Institute of Cardiovascular Science, London, United Kingdom; 2 grid.419255.e0000000446490885Cardiac Modelling Department, Simula Research Laboratory, Oslo, Norway; 3 ASCLEPIOS Project, INRIA Sophia Antipolis-Méditeranée, Sophia Antipolis, France; 4 grid.420468.cCentre for Cardiovascular Imaging, UCL Institute of Child Health, Great Ormond Street Hospital for Children, London, United Kingdom

## Background

Left ventricular ejection fraction (LVEF) late after arterial switch operation (ASO) is often normal. However, some studies have shown increased indexed end diastolic volume (iEDV) and ventricular mass when compared to healthy controls. We sought to identify LV morphological differences between patients with transposition of the great arteries (TGA post ASO) and matched healthy control subjects. We hypothesised that besides a mere increase in ventricular volume, characteristic shape features can be associated with LV morphology post ASO. Ventricular shape being difficult to quantify using traditional morphometrics, a novel, validated non-parametric statistical shape modelling framework (SSM) was used to analyse 3D anatomical models without the need for landmarking.

## Methods

**Figure 1:**
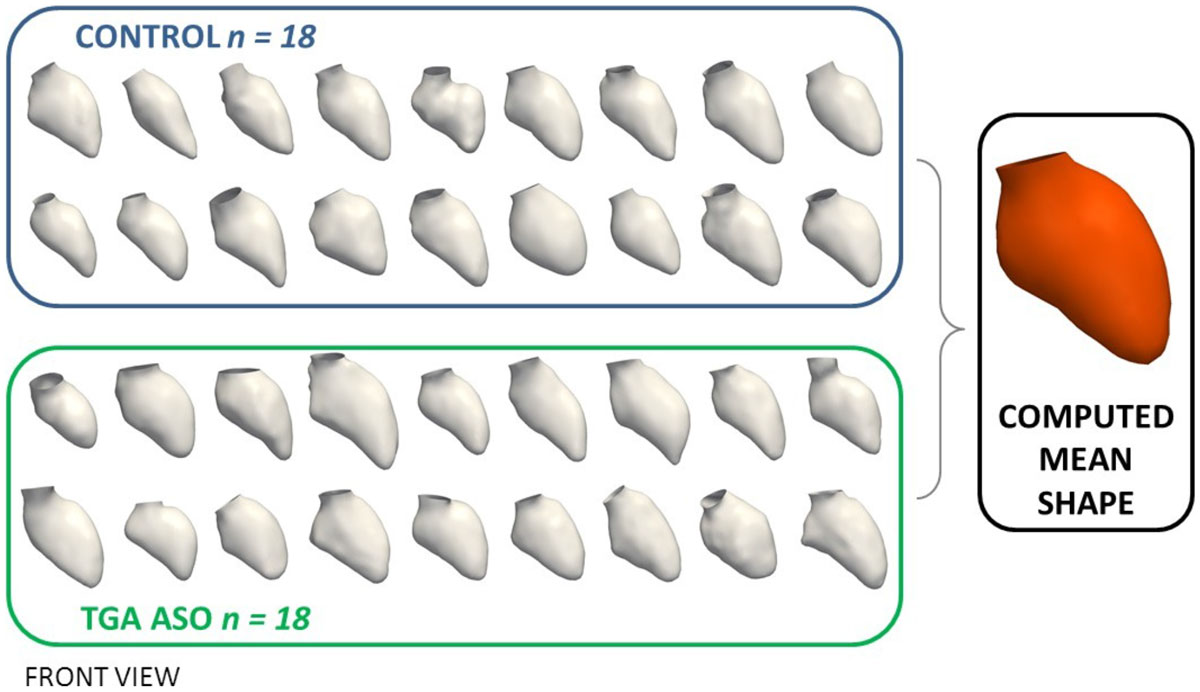
**The 3D LV mean shape was computed using a statistical shape modelling framework based on a population of in total 36 LV shapes reconstructed from CMR data**.

Cardiovascular magnetic resonance (CMR) data from 18 patients post ASO and 18 age-matched healthy control subjects (age 15.0 ± 2.1 years and 15.2 ± 2.0 years, respectively) were analysed. Institutional review board approved the study; all patients/carers signed informed consent. 3D geometric LV surface models were manually reconstructed from the CMR data (3D balanced, steady-state free precession whole heart acquisition) and constituted the input for the SSM. Based on the 36 LV input shapes, the mean LV shape and its deformations towards each patient anatomy were computed (Figure 1). Partial least squares regression (PLS) was applied to the full set of deformation vectors to extract a *shape vector*, which numerically describes 3D shape features most associated with either of the groups. Size effects due to differences in BSA were removed. Results were visualised by deforming the computed mean LV shape along the shape vector from low values for the control group towards high values for the ASO group. Differences were assessed using non-parametric Mann-Whitney-U test. Correlations between shape features and CMR volumetric data (LVEF, iEDV and zEDV = EDV z-scores) were assessed using Pearson's correlation. Significance was assumed at p < .05.

## Results

**Figure 2:**
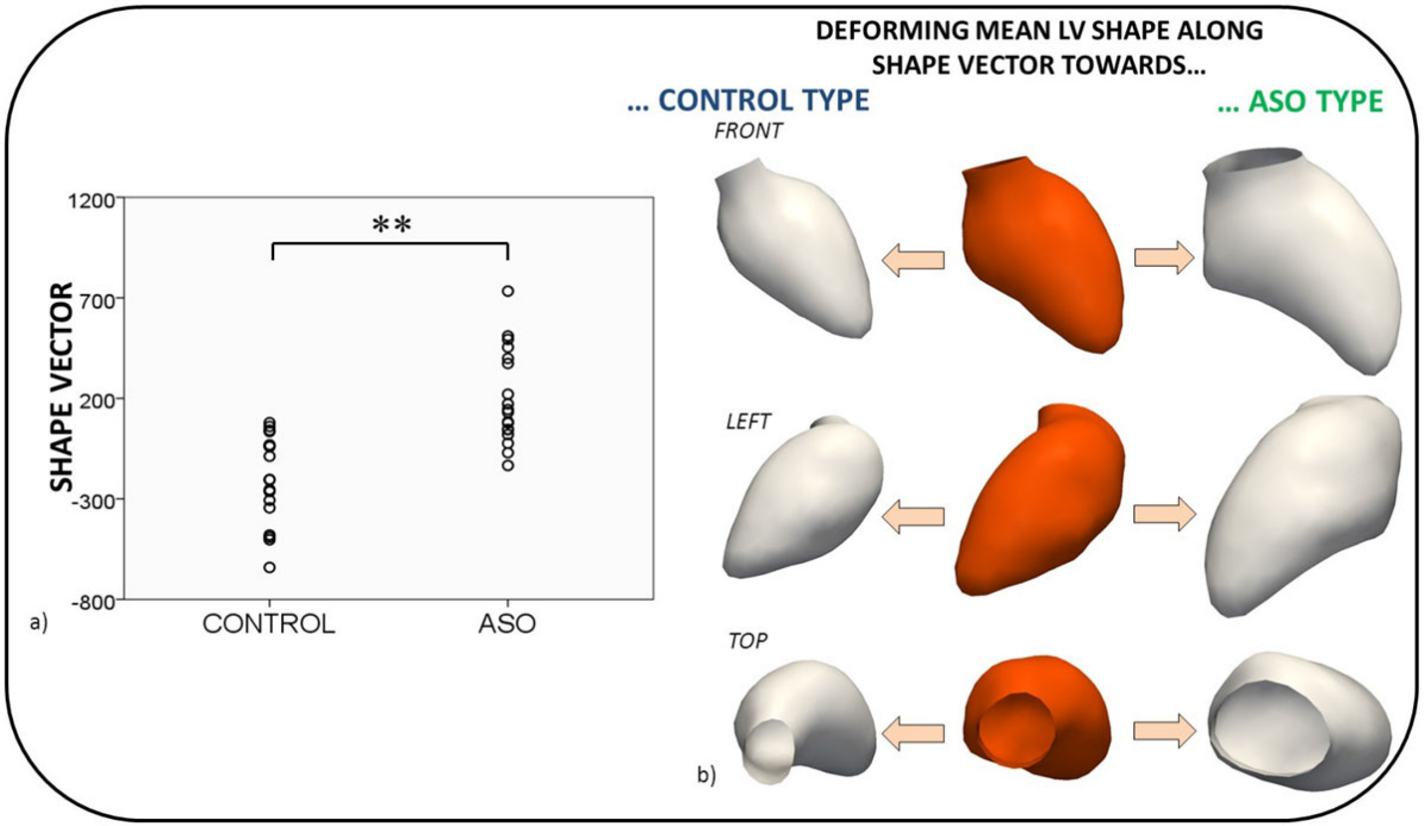
**Comparison of the computed shape vector values for control and ASO subjects suggested significant LV morphological differences between the two groups (a)**. Visualising the results by deforming the computed mean LV shape (b, middle) towards an ASO-like shape revealed an overall enlarged ventricle with a bulged septum and a dilated base being typical for the ASO group (b).

Shape vector values differed significantly between the two groups (p < .001), implying distinct morphological differences in LV shape (Figure 2a). 3D shape visualisation of the shape vector revealed an overall dilated LV with a bulged septum and a dilated base for the ASO group, compared to a rather compact LV for the control group (Figure 2b). ASO-type shape features were not associated with BSA (r = 0.000; p = 1) or LVEF (r = -0.296; p = .080), but correlated with higher iEDV (r = 0.548; p = .001) and zEDV (r = 0.539; p = .001).

## Conclusions

Using a SSM framework based on CMR, distinct 3D shape features could be associated with LV morphology post ASO. Moreover, those shape features were linked with higher iEDV. Such advanced post-processing of CMR data could lead to identifying previously unknown 3D shape features of cardiac anatomy that distinguish healthy and diseased subjects, thereby constituting novel shape biomarkers for improved CMR-based diagnosis.

